# Nitrogen fixation rates and aerial root production among maize landraces

**DOI:** 10.3389/fpls.2025.1502884

**Published:** 2025-01-28

**Authors:** Layne N. Connolly, Nicola Lorenz, Keyvan Maleki, Noah Kayafas, Richard P. Dick, Kristin L. Mercer

**Affiliations:** ^1^ Department of Horticulture and Crop Science, The Ohio State University, Columbus, OH, United States; ^2^ School of Environment and Natural Resources, The Ohio State University, Columbus, OH, United States

**Keywords:** aerial roots, biological nitrogen fixation, nitrogen fertilization, ^15^N natural abundance, mucilage, maize

## Abstract

In Mexico, the center of maize origin (*Zea mays* ssp. *mays*), there are landraces from the highlands that develop extensive aerial root systems which secrete a carbohydrate-rich mucilage. This mucilage produces a favorable environment for nitrogenase activity by diazotrophs. This plant-microbial interaction enables the fixation of nitrogen (N) from the atmosphere, reducing the required N that otherwise must come from the soil and/or fertilizers. The objective of this research was to investigate the degree to which other landraces of maize and nutrient management affect aerial root growth and the ability of maize to perform and benefit from N_2_ fixation. In two replicated field experiments in Columbus, Ohio, USA in 2019 and 2020, we planted 21 maize landraces and three improved varieties with and without fertilizer to measure their growth, production of aerial roots, and rate of atmospheric N_2_ fixation using the ^15^N natural abundance method. Maize accessions varied in the growth rate and number of nodes with aerial roots. Up to 36% of plant N was derived from the atmosphere, with values varying by accession, the reference plant used, and the fertilizer level. Moreover, there was a positive relationship between early growth parameters and numbers of nodes with aerial roots, which, in turn, predicted the amount of N derived from the atmosphere. Thus, larger seedlings may experience enhanced root growth and thereby benefit more from N fixation. By phenotyping a diverse set of maize accessions with and without fertilizer, this study explores both environmental and quantitative genetic variation in the traits involved in N fixation capacity, clarifying that N fixation found in the Sierra Mixe landrace is more broadly distributed than previously thought. In sum, farmers stewarding genetic diversity in a crop center of origin have preserved traits essential for biological symbioses that contribute to maize's nutrient requirements. These traits may enable maize crops grown by Mexican farmers, and farmers globally, to benefit from N fixation from the atmosphere.

## 1 Introduction

Maize (*Zea mays* ssp. *mays*), known for its tall stature and high productivity, has high nitrogen (N) demands, requiring more N than many other staple crops, including rice (*Oryza sativa* L.) or wheat (*Triticum aestivum* L.) (Liu et al., 2006; Buresh et al., 2010; Sheoran et al., 2021). The N demand of maize is commonly met with synthetic fertilizers (Fernández et al., 2009), which utilize a large amount of energy in manufacturing and transport (estimated at 1.2% of the global energy supply) (Heffer and Prud’homme, 2016). Nitrogen fertilizers also have major environmental impacts, including N losses to ground and surface waters causing eutrophication of aquatic ecosystems (Glendining et al., 2009; Mahmud et al., 2020); creation of reactive N species that contribute to stratospheric ozone depletion (Kanter, 2018); and emission of greenhouse gases (2.1% of global production) (Menegat et al., 2022). Thus, alternative N fertilization systems are needed to reduce or eliminate the need for synthetic N fertilizer for the major grain crops, including maize.

Biological N fixation (BNF), whereby diazotrophic bacteria that can closely associate with non-legumes fix atmospheric N_2_ for the plant, has the potential to meet this challenge. Free living diazotrophs that can provide fixed nitrogen to non-legumes exist in all soils (Bloch et al., 2020). In fact, there is a long history of research to explore and optimize BNF in the rhizo- and endo-sphere of maize, sorghum, rice, and sugar cane (Baldani et al., 1986; Döbereiner, 1992; Triplett, 1996; Montañez et al., 2007; Roesch et al., 2008; Keyeo et al., 2011; Bloch et al., 2020; Zhang et al., 2022; Venado et al., 2023). Unfortunately, except for sugar cane (*Saccharum officinarum* L.), where there can be a significant amount BNF (Boddey et al., 2003), the rates of BNF for cereal crops have generally been very low (Pankievicz et al., 2019). Raising those rates may require a targeted approach.

Plants with little access to soil N might be more likely to participate in such an association (Wen et al., 2021), as found in legumes (Taylor and Menge, 2018). If so, we might expect plant species or accessions within species from low N conditions to excel at associating with and benefiting from diazotrophs—as was seen in sugar cane bred under low N (Baldani et al., 2002). Crop varieties grown in low input agroecosystems, as well as wild relatives growing in natural ecosystems, may then also be promising candidates for participating in similar associations (Wen et al., 2021; Santos et al., 2023). Moreover, researchers have identified variation for microbial communities that associate with crop varieties, indicating that genetic variation may exist for the ability to attract and benefit from N fixing microbes within a crop species (Emmett et al., 2017; Goldstein et al., 2019). Centers of crop origin, which contain impressive diversity of crop wild relatives and landraces (i.e., traditional varieties) grown by farmers under low N conditions, may then be promising sources of crop genetic resources getting N from BNF.

Recently, a maize landrace from the highlands of Oaxaca, Mexico, was found to have received up to 82% of its N from BNF (Van Deynze et al., 2018). This landrace harbored diazotrophic bacteria in the mucilage on its aerial roots, thereby feeding the microbes with polysaccharides and providing an oxygen depleted environment that encouraged BNF (Pozzo et al., 2018; Van Deynze et al., 2018). Mexico, the center of crop origin for maize, contains many named landraces grown by farmers (Wellhausen et al., 1952), some of which had previously been found to participate in associations with N fixing diazotrophic microbes (Estrada et al., 2002). This novel phenomenon identified by Van Deynze et al. (2018) has ignited further exploration of the production of aerial roots and mucilage that support N fixation in maize (Bennett et al., 2020; Higdon et al., 2020; Pankievicz et al., 2022; Gao et al., 2023). Nevertheless, we do not yet understand the degree to which other Mexican landraces share the characteristics, such as many large diameter aerial roots, that could benefit this association (but see Pankievicz et al., 2022 and Goldstein, 2015). Nor do we know if soil N might enhance or reduce BNF on maize aerial roots.

A note on the variable nomenclature for aerial roots: They are sometimes called aerial roots (e.g., Van Deynze et al., 2018; Zhang et al., 2018; Pankievicz et al., 2022) and sometimes aerial brace roots (Blizard and Sparks 2020; Reneau et al., 2020). Most basically, they are nodal roots since they grow from the nodes along a plant stem. Nodal roots that grow into the soil are often called brace roots, especially in corn (Sparks, 2023). For the purposes of this article, we will refer to nodal roots above ground as aerial roots, and we will note when these aerial roots may also be rooted and function as brace roots.

Therefore, the objective of this study was to determine the impacts of N management and landrace diversity on aerial root phenology and abundance, plant growth, and N_2_ fixation (using the ^15^N natural abundance method). We also aimed to assess relationships between aerial root, plant growth, and N_2_ fixation traits. To address this, two common garden experiments were established in Ohio using maize landraces from Mexico and South America, as well as improved varieties, with and without added N fertilizer. The hypotheses were that the landraces would vary in aerial root production and N_2_ fixation, showing more of both than improved varieties. Moreover, we expected that N_2_ fixation by diazotrophs would be higher under lower N conditions. Identifying germplasm with the ability to fix N_2_ from the atmosphere will facilitate the preservation and use of related traits from crop centers of origin. These expected outcomes would provide a foundation for development of more sustainable maize cultivation by reducing the reliance on fossil fuel-intensive N fertilizers and mitigating the adverse effects of these fertilizers on soil, water, and environmental quality.

## 2 Materials and methods

### 2.1 Genetic materials

We performed two field experiments (2019 and 2020) at the Ohio State University Waterman Agricultural and Natural Resources Laboratory (WANRL) in Columbus, Ohio (40°00'32.4"N 83°02'14.5"W, 226 masl) to assess highland maize landrace accessions from Mexico for traits related to N fixation. Highland Mexican landraces were emphasized since the landrace from Van Deynze et al. (2018) was also from the highlands. In 2019, we compared 16 accessions of maize, 13 of which were landraces from highland areas (>1800 masl) of Mexico (**Supplementary Table S1**). Other accessions included a landrace from Chiapas, Mexico from a mid-elevation (~1550 masl); an outcrossing US variety; and a low elevation Mexican inbred line (176 masl) (**Supplementary Table S1**). In 2020, we compared nine accessions of maize and sunflower. Six of the eight maize landraces were highland Mexican accessions (> 2000 masl), one was a Mexican midland landrace (1413 masl), and one was from the lowlands (223 masl) in Peru (**Supplementary Table S1**). The sunflower accession was an improved, non-GMO hybrid. These last two accessions were included as putatively non-N_2_ fixing reference accessions. Weather averages for WANRL and locations of origin of maize accessions for the 2019 and 2020 growing seasons were accessed via the weather station located on the farm (for WANRL) and through the Worldclim database using the *raster* package (Hijmans and van Etten, 2016) in the R software environment (ver. 4.2.1) (R Core Team, 2022) for the locations of origin of maize accessions (**Supplementary Table S2**).

### 2.2 Experimental design and management

In 2019, the experiment compared accessions and had a randomized complete block design with the 16 accessions randomized within each of the five blocks. We planted using the traditional Mexican method of three seeds per planting location (i.e., per *mata*). Each plot consisted of two adjoining *matas* for a total of 6 plants per accession per plot. Matas were spaced 0.6 m apart within the row with 1.0 m between rows and were kept well weeded. Starting at vegetative stage 2 (Iowa State University, 2016), each plant was marked to differentiate experimental plants within a *mata*. No irrigation was applied. In 2020, the experiment compared accessions with and without a fertilizer treatment using a randomized complete block design with four blocks. The experiment had a completely randomized split-plot design (four blocks) with fertilizer as the main plot and randomized maize accession as the subplot. The subplots consisted of two short rows (0.75 m between rows) with four plants each (0.2 m spacing within the row) for eight plants per subplot. Directly before planting, N and other nutrients were added in the form of Re-Vita Pro 5-4-5 fertilizer (Ohio Earth Food, OH) (with average δ^15^N of 6.3 (^0^/_00_)), composed of poultry manure, sea kelp, humate, bone meal, and sulfate of potash. This was incorporated into each fertilizer main plot in strips where seeds would be planted, which constituted about two-thirds of the plot. The planted strips had fertilizer applied at a rate of 5.7 kg Re-Vita per 23.7 m^2^ plot x 0.6667, which equated to 0.16 kg m^-2^, or 3,607 kg Re-Vita ha^-1^ (equivalent to 180.35 kg N ha^-1^, 144.28 kg P ha^-1^, and 180.35 kg K ha^-1^). The site previously had alfalfa (*Medicago sativa* L.) growing in the area. Two to three times per week the plots were irrigated, except when >1.3 cm of rainfall occurred in the previous 24 hours. Plots were weeded manually.

### 2.3 Data collection: roots, height, and diameter

In 2019, initial data was taken at 27 days after planting (DAP), including height from the base of the plant to the collar of the newest fully extended leaf, width of the broadest part of the newest fully extended leaf, and diameter of the main stem at approximately 5 cm above the ground. The number of aboveground nodes with roots was also recorded; this trait was observed twice per week for the rest of the experiment. To be counted as an aboveground node with roots, nodal roots had to have broken through the outer layer of the stem (**Supplementary Figure S1**). The first two aboveground nodes with roots (often at or slightly above the soil surface) were not counted since they were considered brace roots, not aerial roots; this counting strategy changed in 2020 due to a more uniform method that could be adopted for counting root layers, published by Zhang et al. (2018). At the experiment's end, final height and leaf width were measured again.

In 2020, there were three periods of data collection. The first was conducted in late July (approximately 45 DAP) to capture early growth and characteristics prior to major aerial root production. Plant height, leaf width, and stem diameter were measured as they had been in 2019. In contrast to how the number of aboveground nodes with roots were counted in 2019, a node with roots at or slightly below the soil surface was counted as node zero, and all nodes above that were then sequentially counted (as in Zhang et al., 2018). This change was adopted because it resulted in more uniform counting of aerial nodes as compared to the 2019 data collection, when determining which two aboveground nodes were considered brace roots was somewhat variable. This method better documented actual visible nodes, but, for a given plant, the 2020 approach would have resulted in higher node counts than in 2019. Final data collection in mid-September (92 DAP) measured number of nodes with roots, plant height, and stem diameter. In neither 2019 nor 2020 did we take data on seed production or yield because accessions from Mexico and Peru were being grown well outside of their zone of adaptation. Most importantly, the long days during the growing season in Ohio delayed flowering for the tropical materials, resulting in little or unrepresentatively low levels of seed production that would have had nothing to do with N availability. The performance metrics we did collect, e.g., height, would have been responsive to N fixed prior to our collection of tissue for ^15^N isotopic analysis (see below).

### 2.4 Data collection: ^15^N isotope measurements

In 2020, to assess sources of N utilized by the plant and estimate N fixation, the ratios of N isotopes were measured in maize landraces that produced high numbers of nodes with aerial roots (CIM 8562, CIM 23370, PUEB 402, and MEXI 212) and compared them against those from conventional maize and sunflower. We also included the landrace with the thickest aerial roots (LIMA 13), which had produced a high number of nodes with aerial roots in another garden (K.L. Mercer, personal obs.). Leaf samples taken from three (non-lodged) plants of each accession were bulked in each fertilizer level. The wet weight of the leaves was recorded; then the samples were dried completely at 55°C and dry weight was recorded. Each sample was ground using a Wiley Model 4, 115V plant mill (Thomas Scientific, NJ). To minimize contamination, the mill was thoroughly sanitized by using a vacuum, air compressor, and 80% ethanol after each sample. Each sample was then dried for about one hour at 55°C to remove moisture before 10-20g of tissue was finely ground to pass a 100-mesh size sieve using a Udy cyclone plant mill (UDY Corporation, CO; **Supplementary Figure S2**), sanitizing between samples.

The ^15^N natural abundance method was used to measure the degree to which N in the plant came from fixation of atmospheric N_2_ (Chalk et al., 2019). ^15^N natural abundance is based on the ratios of naturally occurring ^15^N and ^14^N isotopes (δ^15^N ratio = ^15^N/^14^N) in the atmosphere. From the δ^15^N ratio of a focal accession and reference plants, percent of N derived from the atmosphere can be calculated (%Ndfa = [(δ^15^N reference - δ^15^N focal)/ (δ^15^N reference – B)] x 100), where B is the abundance of ^15^N in the air and assumed to be zero.

For ^15^N analysis (as described by Bremer and Van Kessel, 1990), approximately 5 mg plant tissue was combusted (1800°C) using an elemental analyzer (CHN EA 1108, Carlo Erba Instruments, Italy) that produces N oxides (NOx) that were reduced to N_2_. Subsequently, N_2_ was separated using a chromatographic column (Porapak QS® sorbent tube (Restek), PQS) with one part passing a thermal conductivity detector for %N_2_ quantification and a separate portion receiving N isotopic analysis on a Thermo Scientific Delta V Advantage Isotope Ratio Mass Spectrometer (IRMS). ^15^N abundances were expressed as δ^15^N [‰] (Knowles and Blackburn 1993). Natural abundance of δ^15^N [‰] equals [(Rsample - Rstandard) - 1] * 1000 (Mariotti, 1983), where R is the ratio of ^15^N/^14^N (atom %) of the sample and the standard, and the standard is atmospheric N_2_ (0.00366295 ^15^N abundance). Isotopic reference standard was Acetanilide (Schimmelmann et al., 2009), which was used as a calibration standard for %N and ^15^N.

### 2.5 Data analysis

#### 2.5.1 Logistic estimation of number of nodes over time

In 2019, the number of nodes with aboveground roots taken twice a week from 27 DAP was averaged for each accession at each date (**Supplementary Figure S3**). We developed a logistic growth model to predict the emergence of aerial roots along nodes over time for each accession with a similar method as is used for other plant traits, such as seed germination or seedling growth. Growth models can quantify growth patterns (Paine et al., 2012; Lamont et al., 2023) and estimated parameters can provide insight into growth features of plants. To obtain growth curves for nodal roots of each accession, the following equation was used (Paine et al., 2012):


$$F\left(x\right)=\frac{{\text{Y}}_{M}\times Y_{0}}{\left({\text{Y}}_{M}-{\text{Y}}_{0}\right)\times \text{exp}(-\text{k }\times {\text{x) + Y}}_{0}}$$

where Y refers to the number of aerial nodes at time x; Y_M_ (maximum number of nodes) denotes the maximum potential number of aerial nodes for each accession included in this study; k is the growth rate of aerial nodes, which is specific to each accession; Y_0_ (the initial number) is the initial number of nodes at the start of the observation period; and x is the time over which the growth is measured. We averaged the number of aerial nodes from all subsamples (i.e., individual plants) for each accession. The goodness of fit for the model was assessed using the pseudo-R-squared (R^2^) values. All the analysis was performed using the *nls* package implemented within the R environment (ver. 4.2.1) (R Core Team, 2022).

#### 2.5.2 Generalized linear mixed models of growth and N fixation traits

For all analyses of phenotypic response variables, we ran generalized linear mixed models (GLMM) using the *lmer* package in R (ver. 4.2.1) (R Core Team, 2022). In some of these GLMMs, only categorical predictors were used (i.e., an ANOVA approach); whereas the other model also incorporated continuous predictors (i.e., a regression approach). The first GLMM analyzed the 2019 experiment with an ANOVA approach. It included the effects of accession as a fixed factor; block and the block by accession interaction were random effects and accounted for the use of subsamples. The second GLMM analyzed the 2020 experiment and included fertilizer, accession, and their interaction as fixed variables, along with the random effects of block, the block by fertilizer interaction, and the block by accession by fertilizer interaction. These latter two random effects accounted for the split-plot design and subsamples, respectively. We calculated the least squares means and standard errors with the *emmeans* package (ver. 4.2.1) (R Core Team, 2022) and used a Tukey test for mean separations.

A third and fourth GLMM models were used to determine the degree to which plants that grew well early in their life cycle might produce more aerial roots and to which plants with more aerial roots ultimately became larger or fixed more N. This was performed by running simple and multivariate linear regression models to query the relationship between early growth variables predicting aerial root traits and aerial root traits predicting late growth. For the 2019 data, we predicted responses from continuous predictors, including block as a random factor in the model; accession was removed. For the 2020 data, the model used the same fixed variables and random effects mentioned above in the second model; however, continuous predictor variables were added singly and in combination. Early growth variables used to predict final node count included early height (2019, 2020), early stem diameter (2020 only), and early leaf width (2020 only). Final node count was used to predict final leaf width, final height, final stem diameter, %N, δ^15^N, and Ndfa (using both sunflower and conventional maize for references) (2020 only). Early height was also used to predict final height in both years. For all analyses, replanted plants were not included, and those that had either died or lodged during the growing season were also removed.

## 3 Results

### 3.1 Nodal root growth patterns

**Figure 1:**
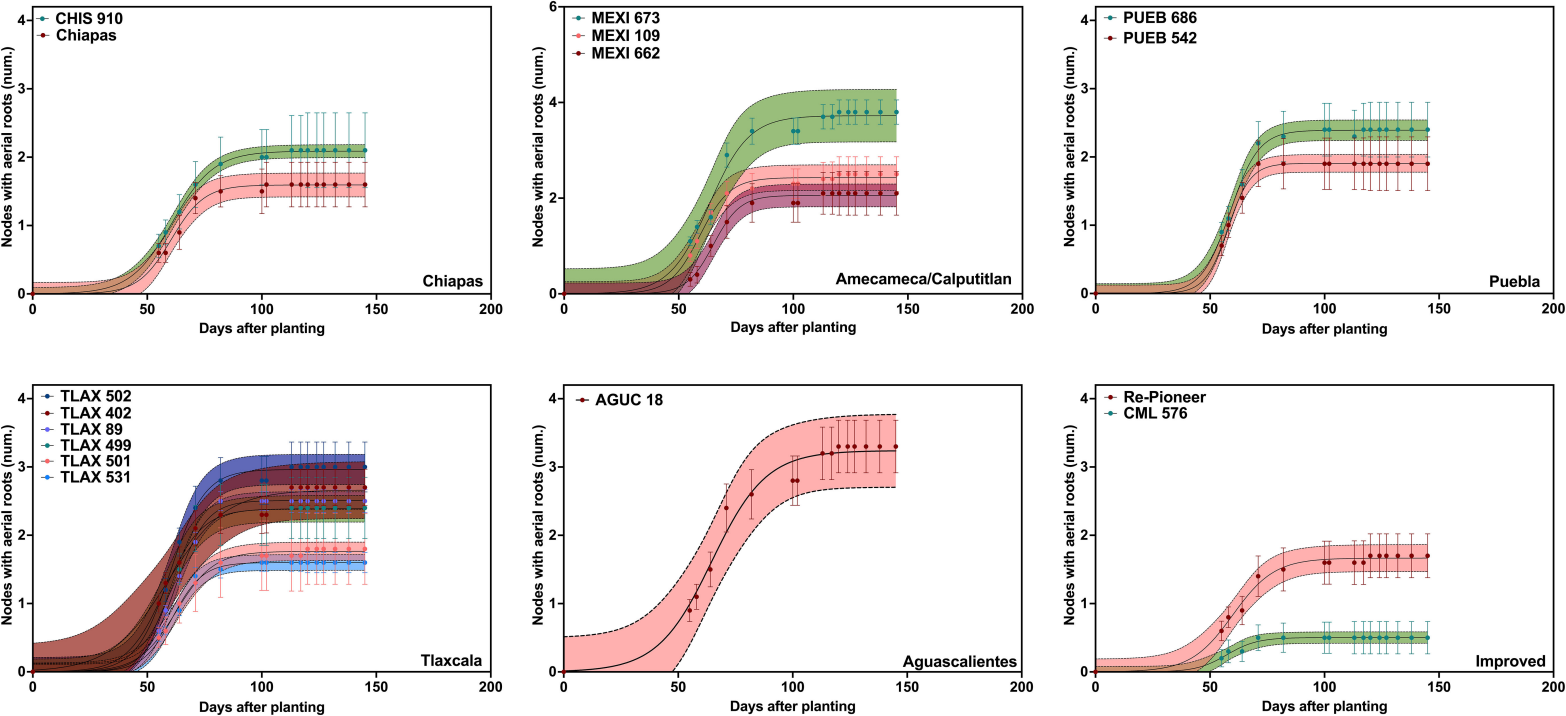
Number of nodes with aerial roots grouped by region of origin grown in the 2019 field experiment at Waterman Agricultural and Natural Resource Laboratory in Columbus, OH. Points represent the average number of aerial nodes from all subsamples (i.e., individual plants) within each plot, while the line shows the fitted values. The shaded areas represent the confidence intervals (CI) of the fitted lines, estimated using the residual standard error from the model applied to the averaged values. Most accessions produced aerial nodes from around 55 days after planting (DAP) and until around 82 DAP. Not all accessions from the same state produced a similar number of nodes. The number of nodes did not include the two lowest nodes that were at or slightly above the soil surface; this resulted in lower averages per accession when compared to the 2020 experiment. See **Supplementary Table S1** for passport data for the accessions.

In 2019, based on the logistic growth models, nodal root growth parameters varied considerably among accessions. The average maximum number of nodes with aerial roots (Y_M_) was 2.2 (**Figure 1**; **Table 1**). MEXI 673 and AGUC 18 showed the highest maximum number of nodal roots (Y_M_), with values of 3.7 and 3.2, respectively, followed by TLAX 502 and TLAX 402, at 2.7 and 3.0, respectively (**Figure 1**; **Table 1**) (it should be noted that the two first nodes with roots were not counted in 2019, so these counts are underestimates relative to what was found in 2020). Conversely, CML 576 (an improved line) and Chiapas had the lowest maximum number of nodal roots (Y_M_), with values of 0.5 and 1.6, respectively (**Figure 1**; **Table 1**). Similarly, there were significant differences in growth rate of nodal roots across landraces (**Figure 1**; **Table 1**). PUEB 542 and MEXI 662 had the highest growth rates, at 0.20 and 0.17 nodes d^-1^, respectively. In contrast, the lowest growth rates were for TLAX 402 and AGUC 18, with rates of 0.08 nodes d^-1^ for both.

**Table 1:** Parameter values for logistic model estimating the addition of aerial nodes of maize landraces grown in Columbus, Ohio, over time.

Accession	Maximum number of aerial nodes (Y_M_)	Growth rate of aerial nodes (k-day^-1^)	Growth duration (days)	Pseudo-R^2^
PUEB 686	2.39 ±0.03	0.15 ±0.02	57.64 ±1.21	0.99
Chiapas	1.59 ±0.01	0.13 ±0.03	59.80 ±0.90	0.98
Re-Pioneer	1.66 ±0.04	0.10 ±0.03	59.52 ±0.98	0.98
TLAX 499	2.38 ±0.09	0.12 ±0.01	57.13 ±0.66	0.99
PUEB 542	1.90 ±0.02	0.20 ±0.02	54.73 ± 0.76	0.99
CML 576	0.50 ±0.01	0.15 ±0.02	55.80 ±0.02	0. 97
MEXI 673	3.72 ±0.12	0.11 ±0.03	63.38 ±1.42	0.99
CHIS 910	2.08 ±0.02	0.10 ±0.01	60.80 ±0.36	0.99
TLAX 501	1.76 ±0.03	0.12 ±0.01	63.87 ±0.03	0.99
TLAX 502	2.96 ±0.04	0.13 ±0.01	59.55 ±0.50	0.99
AGUC 18	3.24 ±0.19	0.08 ±0.04	62.24 ±0.02	0.97
TLAX 402	2.66 ±0.15	0.07 ±0.02	59.69 ±1.47	0.97
MEXI 109	2.43 ±0.05	0.15 ±0.04	58.64 ±0.58	0.98
TLAX 89	2.50 ±0.02	0.14 ±0.01	62.86 ±0.41	0.99
MEXI 662	2.05 ±0.05	0.17 ±0.03	64.51 ±0.01	0.99
TLAX 531	1.60 ±0.02	0.15 ±0.01	61.97 ±0.47	0.99
**Average**	**2.21 ±0.11**	**0.13 ±0.00**	**60.13±0.724**	
**Standard deviation**	**0.190**	**0.008**	**0.724**	

Growth rate of nodes with aerial roots across landraces varied, and the maximum number and growth rate of nodes with roots had a negative relationship, suggesting that plants with more aerial nodes exhibit a slower development rate of those nodes. Parameter values ± standard error. The parameters were estimated using the logistic model. Each parameter value represents the estimated mean across four replicates, with corresponding standard errors (SE). Bold numbers indicate averages and standard deviations for the whole dataset.

Interestingly, there was a negative relationship between the maximum number of nodes with aerial roots and the growth rate of these nodes with roots. This suggests that plants with a higher number of aerial nodes exhibit a slower development rate of aerial nodes compared to plants with fewer aerial nodes with roots (**Supplementary Figure S4**; note that growth rate of aerial nodes refers to each node with aerial roots, not the length of each root on a node). Most accessions started producing aerial nodes around 55 DAP and continued to develop roots at a steady rate until around 82 DAP. Then accessions with higher numbers of nodes continued to add nodes with aerial roots. Not all accessions from the same state produced a similar number of nodes (**Figure 1**). For instance, TLAX accessions from the Mexican state of Tlaxcala, had a range of numbers of aerial nodes with roots. Growth duration varied among accessions, with MEXI 662 (64.5 d) and TLAX 501 (63.9 d) having longer growth duration, while PUEB 542 (54.7 d) and CML 576 (55.8 d) showed shorter growth duration (**Table 1**). Although mucilage was not quantified, it was clearly observable on aerial roots, especially after irrigation or rain events (**Supplementary Figure S1**).

### 3.2 Generalized linear mixed models

**Figure 2:**
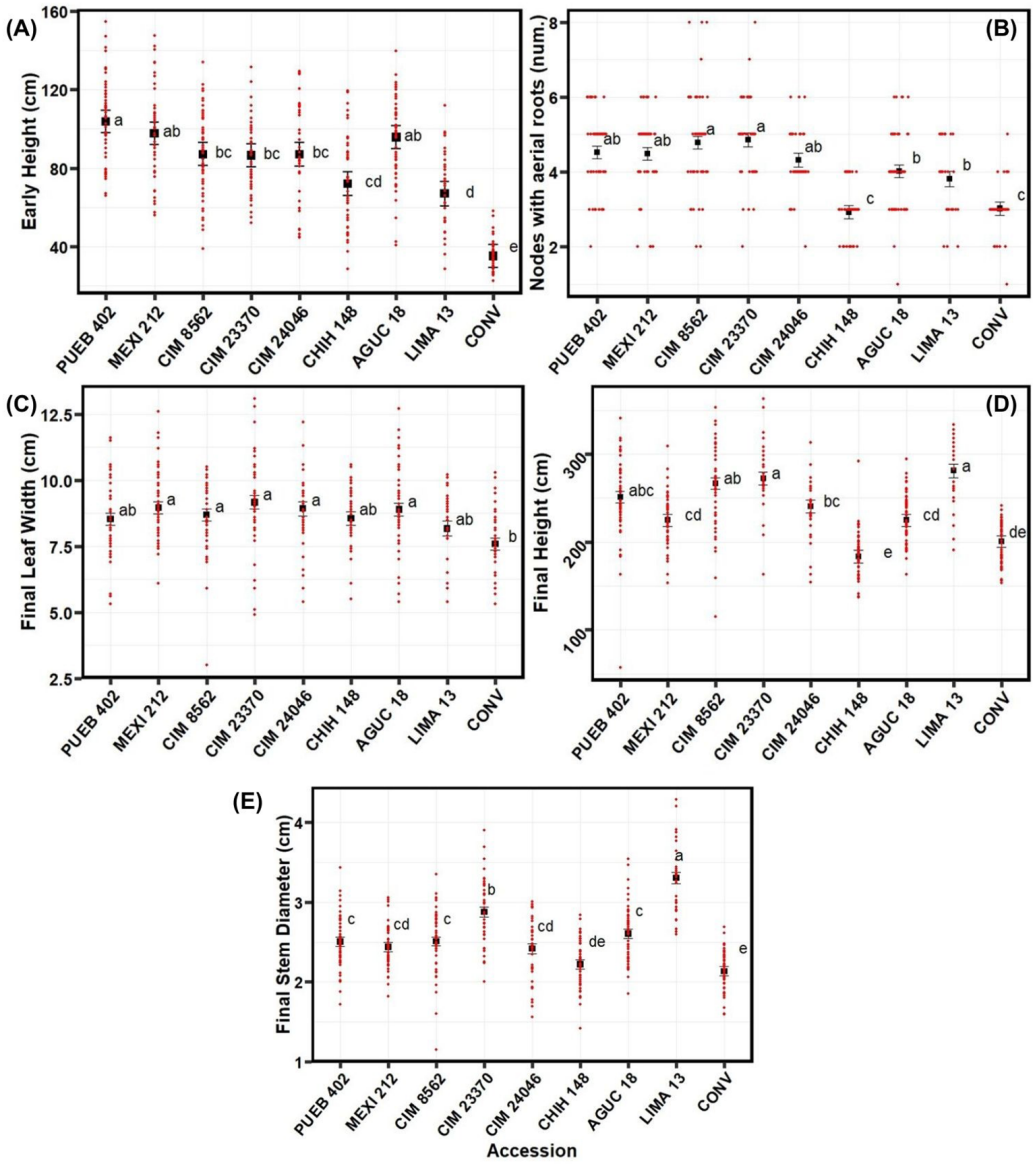
Mean separation of phenotypic traits by accession for the 2020 experiment conducted at Waterman Agricultural and Natural Resources Laboratory, Columbus, OH, USA, including **(A)** early height, **(B)** number of nodes with aerial roots, **(C)** final leaf width, **(D)** final height, and **(E)** final stem diameter. Accessions are ordered by area of origin. See **Supplementary Table S1** for passport data for the accessions. Black data points represent least squares means & error bars indicate the standard error. Red data points show actual measured values across all four blocks and fertilizer treatments. All traits showed significant variation across accessions. Means marked with the same letter are not significantly different using the Tukey test (p<0.05).

The difference among accessions for the final number of nodes with aerial roots was significant in 2020 and close to significant in 2019 (**Table 2**). The high replicate variability in node counts within a particular accession in 2019 may have precluded significance with the GLMM that was obscured in the logistic growth curve analysis above. All other traits were significantly affected by accessions in both years (although final stem diameter was not analyzed in 2019; **Table 2**; **Figure 2**; 2019 mean separation not shown).

**Table 2:** ANOVA results from the GLMM analysis of multiple traits of maize grown in Columbus, Ohio, using the fixed variables fertilizer, accession, and their interaction.

Source of variation	Early Height	# of Aerial Nodes	Final Leaf Width	Final Height	Final Stem Diam
2019					
**Accession**	4.91 _15,36_***	^§^1.65 _15,47_~	1.99 _15,43_*	^¥^2.11 _15,45_*	NA
2020					
**Fertilizer**	1.50 _1,3_^ns^	0.40 _1,3_^ns^	3.92 _1,6_~	0.95 _1,6_^ns^	4.29 _1,3_^ns^
**Accession**	23.53 _1,38_****	16 _8,47_****	4.16 _8,39_***	11.48 _8,47_****	19.60 _8,39_****
**Fertilizer x Accession**	1.70 _1,37_^ns^	1.00 _8,46_^ns^	1.19 _8,39_^ns^	0.93 _8,46_^ns^	1.81 _8,38_ ~

F-value_numerator df, denominator df_.

p ≤ 0.0001 = ****, p ≤ 0.001 = ***, p≤ 0.05 = *, p ≤ 0.1 = ~, p>0.1 = ns.

§ square root transformation; ¥ log transformation.

Fertilizer was not applied in the 2019 experiment. We identified differences among accessions for many traits in both years, including the final number of nodes with aerial roots in 2020.

In 2020, the conventional variety (CONV) was significantly shorter than any of the other accessions early in the season; similarly, LIMA 13 was shorter than most accessions (except CHIH 148 and CONV; **Figure 2A**). Interestingly, LIMA 13 came from the lowest elevation of any of the accessions tested in 2020 (223 masl). By contrast, PUEB 402 was the tallest and larger than all other accessions, except for MEXI 212 and AGUC 18 (**Figure 2A**). For final height, the CHIH 148 was the shortest (183 cm), although CONV was not significantly taller (201 cm; **Figure 2D**). LIMA 13 and CIM 23370 were the tallest at the end of the experiment (2813 cm for LIMA 13 and 272 cm for CIM 23370) though they were not significantly larger than PUEB 402 or CIM 8562; **Figure 2D**).

For the number of nodes with aerial roots in 2020, the CONV and CHIH 148 accessions had the lowest number of nodes (with averages of 3.02 for CONV and 2.93 for CHIH 148; **Figure 2B**). CIM 8562 and CIM 23370 had the greatest number of nodes with aerial roots (4.78 for CIM 8562 and 4.86 for CIM 23370; **Figure 2B**), but they did not differ significantly from PUEB 402, MEXI 212, or CIM 24046; **Figure 2B**).

Final leaf width was similar among accessions, but five landraces had significantly (P<0.05) wider leaves than CONV (**Figure 2C**). For the final stem diameter, the CHIH 148 and CONV accessions had the narrowest stems (2.13 cm for CONV and 2.22 cm for CHIH 148; **Figure 2E**), while LIMA 13 had the thickest stems (3.30 cm; **Figure 2E**).

In summary, although LIMA 13 started off small, the conventional variety from the US (CONV) and the landrace from Chihuahua, Mexico (1413 masl, CHIH 148), were the smallest plants at the end of the season, while the rest of the landraces had relatively similar and larger stature.

### 3.3 Nitrogen content analysis results

**Figure 3:**
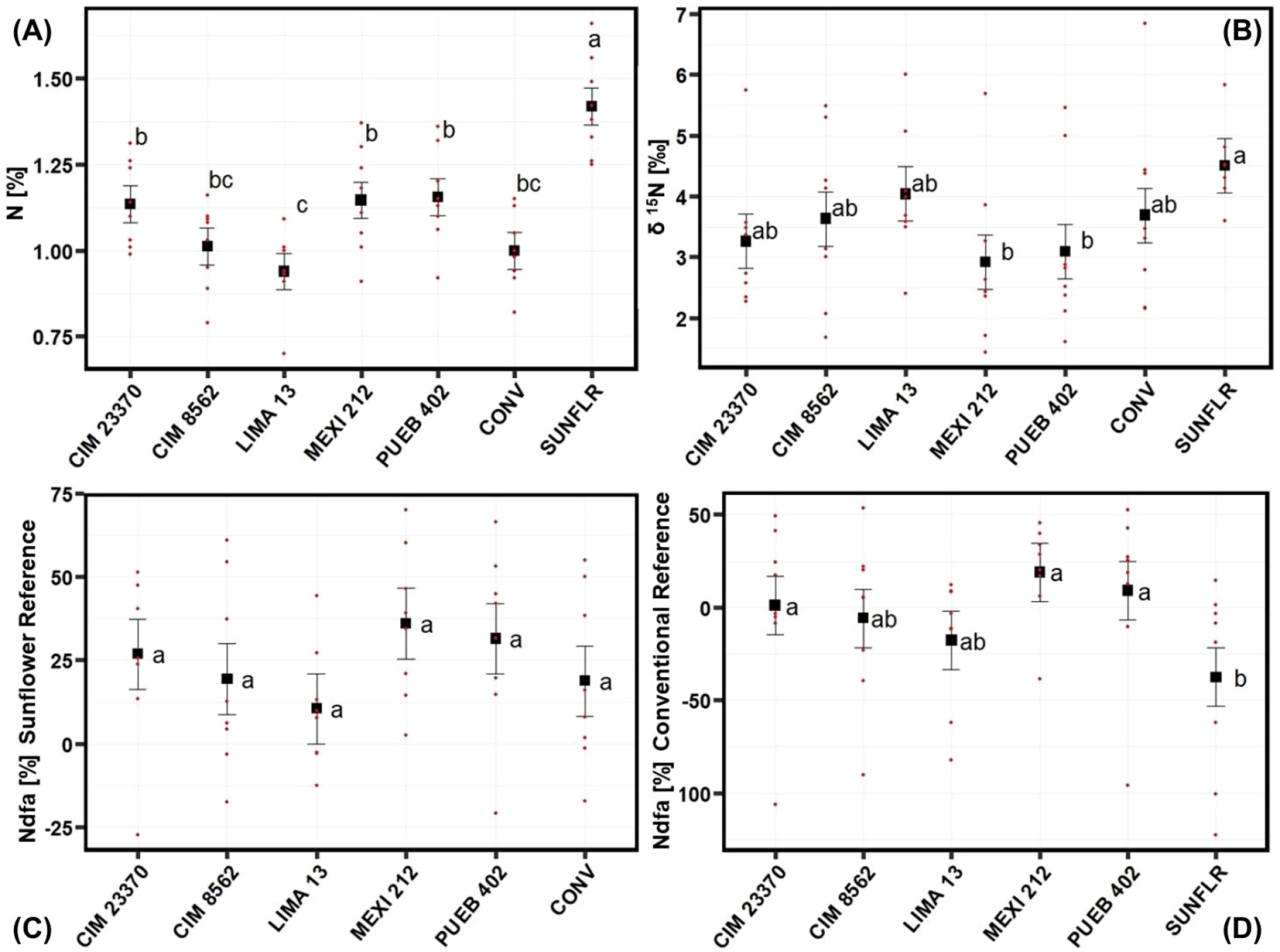
Mean separation by accession for the **(A)** %N, **(B)** δ15N, **(C)** %Ndfa (nitrogen derived from the atmosphere with sunflower reference, and **(D)** %Ndfa with improved maize reference. Data is from the 2020 field experiment conducted at Waterman Agricultural and Natural Resources Laboratory, Columbus, OH, USA. See **Supplementary Table S1** for passport data for the accessions. Black data points represent least squares means and error bars indicate their standard errors. Red data points show actual data points across all four blocks and fertilizer treatments. Means marked with the same letter are not significantly different using the Tukey test (p<0.05). Using sunflower as a reference, Ndfa values were not different among landraces or the conventional variety (CONV), but were larger than zero, indicating the presence of Ndfa in all the maize accessions.

ANOVA of 2020 N data showed that fertilizer significantly increased δ^15^N abundance in leaves (no fertilizer: 2.95; fertilizer: 4.23) and reduced %Ndfa with a sunflower reference (no fertilizer: 33.5%; fertilizer: 14.2%); fertilizer nearly significantly (P = 0.06) increased %N, as well (no fertilizer: 1.14%; fertilizer: 1.09%) (**Table 3**). There was also a significant effect due to accessions for %N, δ^15^N, and natural ^15^N abundance difference (%Ndfa) using the conventional variety (CONV) as a reference (**Table 3**). Sunflower had a higher %N than the maize accessions did and, among maize accessions, CIM23370, MEXI 212, and PUEB 402 had a higher %N than LIMA 13; CIM 8562 and the conventional variety (CONV) were intermediate (**Figure 3A**). Although the sunflower accession had a higher δ^15^N than MEXI 212 and PUEB 402, none of the maize accessions differed from one another (**Figure 3B**). For the %Ndfa using the conventional maize variety (CONV) as a reference, sunflower had the lowest value, but none of the maize landraces differed from one another (**Figure 3D**). %Ndfa values were below zero for CIM 8562, LIMA 13, and sunflower since their δ^15^N values were lower than that of the reference. Interestingly, although there was no accession effect on %Ndfa values using sunflower as a reference (**Table 3**; **Figure 3C**), values ranged from 10.5% (LIMA 13) to 36.0% (MEXI 212) and were larger than zero, including the conventional variety (CONV), indicating the presence of Ndfa in all the maize accessions.

**Table 3:** ANOVA results for N content analysis in maize grown in Columbus, Ohio, in 2020 using the fixed variables fertilizer, accession, and their interaction.

Source of variation	Avg %N	Average δ^15^N	Ndfa (sunflower ref)	Ndfa (improved maize ref)
**Fertilizer**	3.61 _1,39_~	11.31 _1,3_*	14.87 _1,33_***	0.002 _1,3_^ns^
**Accession**	12.83 _6,39_****	2.63 _6,36_*	1.16 _5,33_^ns^	2.98 _4,24_*
**Fertilizer x Accession**	1.48 _6,39_^ns^	0.46 _6,36_^ns^	0.14 _5,33_^ns^	0.22 _4,24_^ns^

F-value_numerator df, denominator df._

p ≤ 0.0001 = ****, p ≤ 0.001 = ***, p≤ 0.05 = *, p ≤ 0.1 = ~, p>0.1 = ns.

Ndfa: percentage of N derived from atmosphere.

The δ^15^N and Ndfa (using sunflower as a reference) were affected by fertilizer and %N, δ^15^N, and Ndfa (using the improved conventional maize as a reference) were affected by accession.

MEXI 212 and PUEB 402 were the maize landraces that tended to have the highest average percent N (other than sunflower) (**Figure 3A**), lowest δ^15^N (**Figure 3B**), highest %Ndfa (**Figure 3C, D**), and they were in the top four for number of aerial nodes produced (**Figure 2B**). LIMA 13 was at the other end of the spectrum for these traits.

### 3.4 Regression analysis

Regression analysis was used to assess the effects of individual continuous predictors. Early height was found to positively predict the final number of nodes with roots in both years. Also, early stem diameter and leaf width also predicted final node count in 2020 (**Table 4**). Thus, plants that were bigger early on produced more nodes with aerial roots. For both years, the final node count also significantly predicted final leaf width, stem diameter, and height (for 2020 final height was nearly significant). These relationships indicate that plants with more nodes experienced greater growth. While our different methods of counting nodes between 2019 and 2020 could have resulted in different slopes for these relationships, we do not see major effects on significance (**Table 4**), although year to year variation may also be involved. Final node count also significantly predicts %Ndfa when using sunflower as a reference (and was close to significant when using improved maize as a reference) (**Table 4**).

**Table 4:** Regression analysis on the relationship between various response variables and their predictor variables in the 2019 and 2020 field experiments.

Response Variable: Final Node Count						
Year	Predictor variable	β	SE	t-value	P	
2020	Early Height	0.018	0.0030	6.9	<0.0001	****
2020	Early Stem Diameter	0.481	0.1240	3.9	<0.0001	****
2020	Early Leaf Width	0.221	0.0350	6.3	<0.0001	****
2019	Early Height	0.085	0.0170	5.0	<0.0001	****
Response Variable: Final Height						
2020	Early Height	0.41	0.10	4.3	<0.0001	****
2020	Final Node Count	3.3	1.8	1.9	0.060	~
2020	Early Stem Diameter	15.27	4.455	3.4	0.00	***
2020	Early Leaf Width	1.7	1.321	1.3	0.196	ns
2019	Early Height	-0.45	0.551	-0.8	0.42	ns
2019	Final Node Count	8.2	1.633	5.0	<0.0001	****
Response Variable: Final Leaf Width						
2020	Final Node Count	0.40	0.065	6.2	<0.0001	****
2019	Final Node Count	1.85	0.440	4.2	<0.0001	****
Response Variable: Final Stem Diameter						
2020	Final Node Count	0.085	0.017	5.1	<0.0001	****
Response Variable: Ndfa (sunflower ref)						
2020	Final Node Count	16.9	7.6	2.2	0.03	*
Response Variable: Ndfa (conventional ref)						
2020	Final Node Count	18.3	10.3	1.8	0.09	~
Response Variable: Avg δ15N						
2020	Final Node Count	-0.71	0.38	-1.9	0.07	~
Response Variable: Avg %N						
2020	Final Node Count	-0.035	0.045	-0.8	0.45	ns

p ≤ 0.0001 = ****, p ≤ 0.001 = ***, p≤ 0.05 = *, p ≤ 0.1 = ~, p>0.1 = ns.

A simple linear regression model was fitted to obtain the coefficients. Plants that were bigger early on produced more nodes with aerial roots. Final node count also predicted final leaf width, stem diameter, and height. These relationships indicate that plants with more nodes experienced greater growth. Final node count also predicts %Ndfa when using sunflower as a reference (and was nearly significant with improved maize as a reference).

A multivariate linear regression analysis simultaneously tested the relationship of multiple parameters with final node count and late height using the 2020 field data (**Table 5**). This showed that, when analyzed a combination of factors, early height and early leaf width were both significant in predicting final node count, while early stem diameter no longer was, likely because variation in stem diameter was explained by height and/or leaf width (**Table 5**). When predicting late height, the multivariate linear regression analysis showed that early height and early stem diameter significantly predicted late height, but final node count (which had been nearly significant) and early leaf width did not (**Table 5**).

**Table 5:** Multivariate linear regression analysis results showing the relationships between various maize traits in 2020 in Columbus, Ohio.

Multivariate Regression Analysis: 2020 Only					
Predictor variable	β	SE	t-value	P	
Response variable: Final Node Count					
Early Height	0.013	0.003	4.423	<0.0001	****
Early Stem Diameter	0.145	0.128	1.133	0.26	ns
Early Leaf Width	0.127	0.040	3.139	0.0018	**
Response variable: Late Height					
Final Node Count	0.980	1.854	0.528	0.60	ns
Early Height	0.359	0.110	3.272	0.0012	**
Early Stem Diameter	14.537	4.831	3.009	0.0028	**
Early Leaf Width	-2.100	1.529	-1.383	0.168	ns

p ≤ 0.0001 = ****, p ≤ 0.01 "**", p ≤ 0.1 = ~, p>0.1 = ns.

Early height and early leaf width both predicted final node count, while early stem diameter did not. Early height and early stem diameter predicted late height, but final node count and early leaf width did not.

## 4 Discussion

This multi-year field experiment showed that there was a significant effect of maize landraces on the number and rate of nodes with aerial roots, as well as evidence that diverse maize landraces (and perhaps improved varieties) are benefiting from atmospheric N_2_ fixation. It was found that larger maize seedlings produced a greater number of nodes with aerial roots and that the number of nodes with aerial roots predicted the amount of N plants derived from atmospheric N_2_ (%Ndfa) in maize landraces. Interestingly, N fixation from the atmosphere was shown to be occurring in Mexican landraces other than those previously studied (e.g., Van Deynze et al., 2018; Pankievicz et al., 2022; Gao et al., 2023), and our results affirm the importance of aerial roots in this process. Finally, we also found some evidence of fertilizer reducing fixed N (%Ndfa) in these landraces.

### 4.1 Development of nodes with aerial roots

While it is known that that growth varies among plant species and even among populations of the same species (Gardarin et al., 2016), some traits are better studied than others. Little is known about the patterns of aerial root growth over time in diverse maize accessions (Sparks, 2023); our study sought to add to the current knowledge by observing the timing of the emergence of aerial roots from nodes over the growing season. Our findings showed that the pattern of addition of nodes with aerial roots varies among a diverse set of maize landraces, especially the maximum number and growth rate of nodes. Various developmental, genetic, or environmental (i.e., biotic or abiotic) factors control the phenology and degree of aerial root growth in plants. While high light and warmer temperatures can trigger nodal root emergence in maize (Tardieu and Pellerin, 1991; López-Frías et al., 2011; Blizard and Sparks, 2020) and related mucilage production also responds to environmental conditions (Pankievicz et al., 2022), aerial root growth is known to also have a genetic basis (Blizard and Sparks, 2020). It is likely that the landraces we studied were variable at genetic loci relevant to aerial root production; since aerial root growth did not respond to fertilizer, we did not see evidence of a clear environmental impact on root growth. However, larger plants did seem to have more aerial nodes, so our fertilizer treatment may not have been strong enough to boost aerial root growth or other nutrients may have been limiting aerial root growth. A better understanding of environmental and genetic factors affecting aerial root growth development could improve our ability to enhance benefits from N fixation.

The aerial node development accelerated from about 55 to 82 DAP. This may support the hypothesis that the plant is putting on aerial nodal roots to supplement increasing N needs of maize. Nitrogen needs have been shown to drastically increase at the V10 growth stage, a critical period characterized by a surge in vegetative growth (Bender et al., 2013). As the plant progresses to the reproductive stages, particularly after the silking stage (R1), N remobilization becomes crucial, transferring N from vegetative tissues to developing grains, ensuring sufficient nutrients for grain filling and maximizing yield efficiency (Freeman et al., 2007; Bender et al., 2013). Further investigation is needed to determine the timing of N uptake of maize landraces over time and how this may correlate to timing of the plant putting on nodes with aerial roots. Notably, N uptake patterns of maize follow a sigmoidal trend (S-shaped) with more than half of the N acquisition occurring at the VT/R1 stages (Bender et al., 2013). While we took no explicit stage data in this experiment, comparison to other experiments using growing degree days clarifies that plants in this experiment would have been expected to be at V10-R1 by 82 DAP. It is significant to find that after 82 DAP, additional aerial nodes were rarely added, even though N uptake of maize plants continues to increase from the V10 to V12 stages. This may indicate that the established nodes with aerial roots and the attending microbial community could continue to benefit the plant as it continues its demand for N. Clarifying the degree to which N fixed from the atmosphere, in part on aerial roots, is contributing to plant N at these late stages would provide valuable insights into the overall picture of maize nutrition acquisition, but would require a separate, detailed investigation.

### 4.2 Nitrogen fixation occurring in diverse maize accessions

Previous research on N_2_ fixation on maize aerial roots has worked with a limited array of genetic materials. As far as we are aware, our study included landraces from the most diverse range of locations across Mexico studied to date; it also includes one South American landrace (**Supplementary Figure S5**). Although, we should not forget about others who have studied landraces and their diazotrophic associations with N_2_ fixing bacteria taking place endophytically or in the rhizosphere (e.g., Estrada et al., 2002; Goldstein et al., 2019). Other studies exploring aerial roots and N fixation have focused on landraces from Oaxaca (Van Deynze et al., 2018; Pankievicz et al., 2022), as well as teosinte and maize inbred lines (Pankievicz et al., 2022; Gao et al., 2023), reaffirming the capacity of maize to fix N and the role of aerial roots and mucilage in that process. Their work and ours have determined that symbiosis with diazotrophic bacteria in mucilage on aerial roots can be generalized past the landrace originally studied to other landraces, maize inbred lines, and teosinte (Pankievicz et al., 2022; Gao et al., 2023; Guo et al., 2023). To expand upon research already conducted in this field, our study investigated whether the addition of fertilizer significantly affected N_2_ fixation and whether there were significant differences among the diverse set of landraces and improved varieties grown.

Using the natural abundance method and comparing ^15^N and ^14^N with that of the non-fixing reference sunflower, we found that unfertilized accessions derived 21-46% of their N from the atmosphere when using sunflower as our reference plant. By contrast, fertilized accessions derived less N from the atmosphere (0-26%) with sunflower as the reference plant, indicating that fertilizer appears to be reducing N fixation in these conditions. The levels of N derived from the atmosphere in our study are in line with findings for the Sierra Mixe landrace of 29-82% N derived from the atmosphere (Van Deynze et al., 2018). Interestingly, when we used a commercial maize hybrid as a reference plant instead of sunflower, N derived from the atmosphere in fertilized accessions was lower than that from unfertilized accessions at 0-16% and 0-22%, respectively. This reduction in apparent N fixation when a commercial maize was used as the reference may indicate that improved varieties may have also maintained some capacity to host and benefit from N fixation. For instance, even though the number of nodes with roots was low in the improved varieties in both years (2019: Re-Pioneer, number of aerial nodes with roots = 1.7; 2020: CONV, number of aerial nodes with roots = 3.02) and lower than most landraces, the CONV variety in 2020 appeared to have benefited to some degree from fixed N based on %Ndfa values (about 19% N from the atmosphere). While our assessment of how improved varieties differ from landraces in N fixation is limited due to the paucity of improved varieties in this study, recent findings underscore the presence of genetic variation in N fixation related traits among modern maize inbred lines, specifically in aerial root mucilage production—a trait now understood to be, at least in part, regulated by the gene ZmSBT3 (Gao et al., 2023).

Percent Ndfa can be elevated in several ways, including uptake through the soil of N originally derived from the atmosphere. For instance, residual N from chemical fertilizer in the soil could increase %Ndfa estimates, if taken up through the belowground roots, since industrial fertilizer is produced through industrial N fixation from the atmosphere. Similarly, the plant uptake of organically derived N from degraded legume biomass, such as a legume cover crop, could also increase %Ndfa estimates. Any N fixation happening in the belowground rhizosphere and then taken up by the plant could also skew these numbers. Additionally, while irrigation was implemented in 2020, relative humidity levels in Ohio are still lower than optimal for mucilage production (based on the landraces’ environment of origin), which may have limited %Ndfa via aerial roots. In 2020 the experiment was implemented on a site that had not received chemical fertilizer for many years. However, prior to planting, an alfalfa crop was grown on the plot area, and its biomass was incorporated.

Nonetheless, we found significant differences among Mexican maize landrace traits, confirming that traits related to N_2_ fixation persists within the crop’s center of origin. This may provide possible evidence for a genetically based adaptation that evolved to enhance N acquisition in different environmental conditions, particularly, N-deficient conditions (Li, 2021; Singh et al., 2022; Zhang et al., 2023). The fact that aerial root production persisted across nearly all landraces suggests that aerial roots likely play a key role in maize plant growth and reproduction, other than just for N fixation (Sparks, 2023). For example, brace roots, which are low aerial roots that grow into the soil, may reduce lodging, take up water and nutrients, and host symbiotic relationships (Blizard and Sparks, 2020). Of great interest here, mucilage produced on aerial roots can harbor diazotrophs and other microbes that enhance plant health, such as through production of plant hormones (Lugtenberg and Kamilova, 2009; Yang et al., 2009).

### 4.3 Relationships between aerial roots, growth, and nitrogen fixation

To expand upon previous research, our study investigated the relationship between growth parameters (plant height, stem diameter, and leaf width) and the number of nodes with aerial roots and whether the number of nodes with aerial roots is significant in N_2_ fixation. The final number of nodes with aerial roots significantly predicted the percent of N derived from the atmosphere (%Ndfa with sunflower as a reference), confirming that aerial roots may play a role in fixing N from the atmosphere (Van Deynze et al., 2018). Others have found that plants with more aerial nodes may be putting on larger aerial roots (Yu et al., 2015; Blizard and Sparks, 2020) and that younger and larger diameter roots tend to produce more mucilage than older roots (Pankievicz et al., 2022). Our findings support the hypothesis that additional layers of aerial roots may be providing an environment where greater amounts of N are fixed from the atmosphere. These findings show the potential for maize plants to meet their N needs with less dependence on N fertilizers that are traditionally applied to the soil.

With the addition of growth parameters that were investigated in the study, we were able to look at the relationship between early growth and final number of nodes with aerial roots, a comparison that has not previously been reported. Early season height and leaf width best predicted the final number of nodes with aerial roots. This supports the hypothesis that bigger plants in the early growth stage produce more nodes with aerial roots, which has been observed in the field (K.L. Mercer, personal obs.). However, this result might be seen as contradictory. N fixation is often thought to be most beneficial to, and active in, a plant when N is limited (e.g., when the plant is not growing well). This is supported by dynamics of N_2_ fixation in legumes where more fixation is triggered or enhanced under low N conditions (Xu et al., 2020; Habinshuti et al., 2021; Irisarri et al., 2021). Thus, it might be expected that smaller maize plants, with more to gain from the carbon investment of aerial root and mucilage production, might excel at adding nodes with aerial roots. However, the opposite was found. Here, large seedlings grew into plants that produced more nodes with aerial roots. Stronger plants producing more aerial roots is supported by farmer observations in southern Mexico that there is greater aerial root production on more fertile soils (personal obs., H. Perales). Similarly, Pioneer (2024) found that maize plants with more resources, such as light and nutrients, produce more nodes with aerial roots.

Additionally, because this study measured final growth paraments, we were able to investigate the relationship between these traits and the number of aerial nodes. The finding that more aerial nodes resulted in greater final growth parameters (final height, final leaf width, and final stem diameter) supports the hypothesis that nodes with aerial roots may be contributing in some way to the production of larger plants. Larger plants may also need more brace roots (Hostetler et al., 2022; Rasmussen et al., 2022), triggering growth of roots on nodes along the plant stem. Nevertheless, many of those nodes have roots that remain aerial rather than tethering the plant more firmly to the ground (Blizard and Sparks, 2020; Sparks, 2023). The development of brace roots and higher nodal roots that remain aerial are likely developmentally and genetically linked (Zhang et al., 2018; Hostetler et al., 2021), but further research is needed to understand more of the mechanisms that regulate this process.

### 4.4 Applications for breeding and future needs

The further utilization of N fixation capacity, such as aerial root production and mucilage secretion in Mexican landraces will be most relevant for the farmers and communities stewarding and growing these accessions *in situ*. Our results indicate that accessions other than those studied by Van Deynze et al. (2018) may be providing N fixation benefits within subsistence and low-input farming systems in Mexico and elsewhere. Assessment of an even broader array of landraces from Mexico and throughout the Americas may help us understand where N fixation is most prevalent and mechanisms at work that maximize fixed N for landrace production, such as environmental conditions and cultural practices. Our results can also provide insight for participatory maize breeding programs in Mexico that can readily integrate tropical materials into improved landraces or hybrid varieties. Integrating these traits with climate resilience strategies could also enhance their value, particularly in marginal or low-input systems.

The integration of nitrogen fixation traits into modern maize breeding programs in Mexico and globally presents both opportunities and challenges. While N fixation traits have the potential to reduce reliance on synthetic fertilizers, their utility in high-input agricultural systems may be constrained by the suppressive effect of high soil N levels on BNF. Elevated N availability can inhibit nitrogenase activity, effectively "switching off" the microbial processes critical for BNF, at least in legumes. Our results indicated that fertilizers may modulate N fixation since we saw a reduction in %Ndfa when fertilizer was applied. If fertilizer switches off N fixation in maize, that will present a significant challenge for deploying these traits in conventional, high-yielding systems. Testing the effects of different forms or levels of fertilizers on N fixation may further clarify the prevalence and strength of this dynamic. Moreover, trade-offs, such as resource allocation between N fixation and yield, would need to be carefully understood to ensure overall productivity would not be compromised by introduction of these novel N fixation traits into commercial lines. Advances in genetic mapping and marker development may end up being useful tools for those interested in transferring N fixation traits into elite germplasm while maintaining yield potential, although such strategies would need to be undertaken with attention to international norms for use of germplasm (e.g., Kloppenburg et al., 2024 and response Bennett et al., 2024). Additionally, research must explore strategies to optimize agronomic practices, such as targeted N application or microbial inoculation, that might be most conducive to BNF. Addressing these challenges will require interdisciplinary efforts to balance the expression and functionality of nitrogen fixation traits with the demands of modern breeding and farming systems.

## 5 Conclusion

This study highlights the potential of maize landraces to leverage BNF through aerial roots and mucilage, possibly contributing to more sustainable N management. Significant variation in aerial root growth and phenology was observed among landraces, with larger seedlings producing more nodes and benefiting more from BNF. The positive relationships between early growth traits, aerial root production, and N fixation emphasize the genetic potential of landraces from maize’s center of origin for sustainable cropping systems. Using the ^15^N natural abundance method, we found that atmospheric nitrogen uptake (%Ndfa) varied across accessions, with some performing comparably to highland Mexican landraces like Sierra Mixe. BNF appears to be a widespread trait in diverse Mexican maize germplasm, offering opportunities for crop improvement in low-input systems. These findings underscore the importance of preserving and studying maize diversity to improve environmental sustainability in maize production. Future research should explore N uptake timing, aerial root microbial communities, and the factors regulating root and mucilage development to optimize BNF in maize cultivation.

## Data Availability

The raw data supporting the conclusions of this article will be made available by the authors, without undue reservation.
