# Supplementary material for: Nitrogen fixation rates and aerial root production among maize landraces
Source: Front Plant Sci. 2025 Jan 28;16:1502884. doi: 10.3389/fpls.2025.1502884 (PMC11811074; doi:10.3389/fpls.2025.1502884)
Supplement: Supplementary file 1 [file DataSheet1.docx]

Supplementary Material: Figures


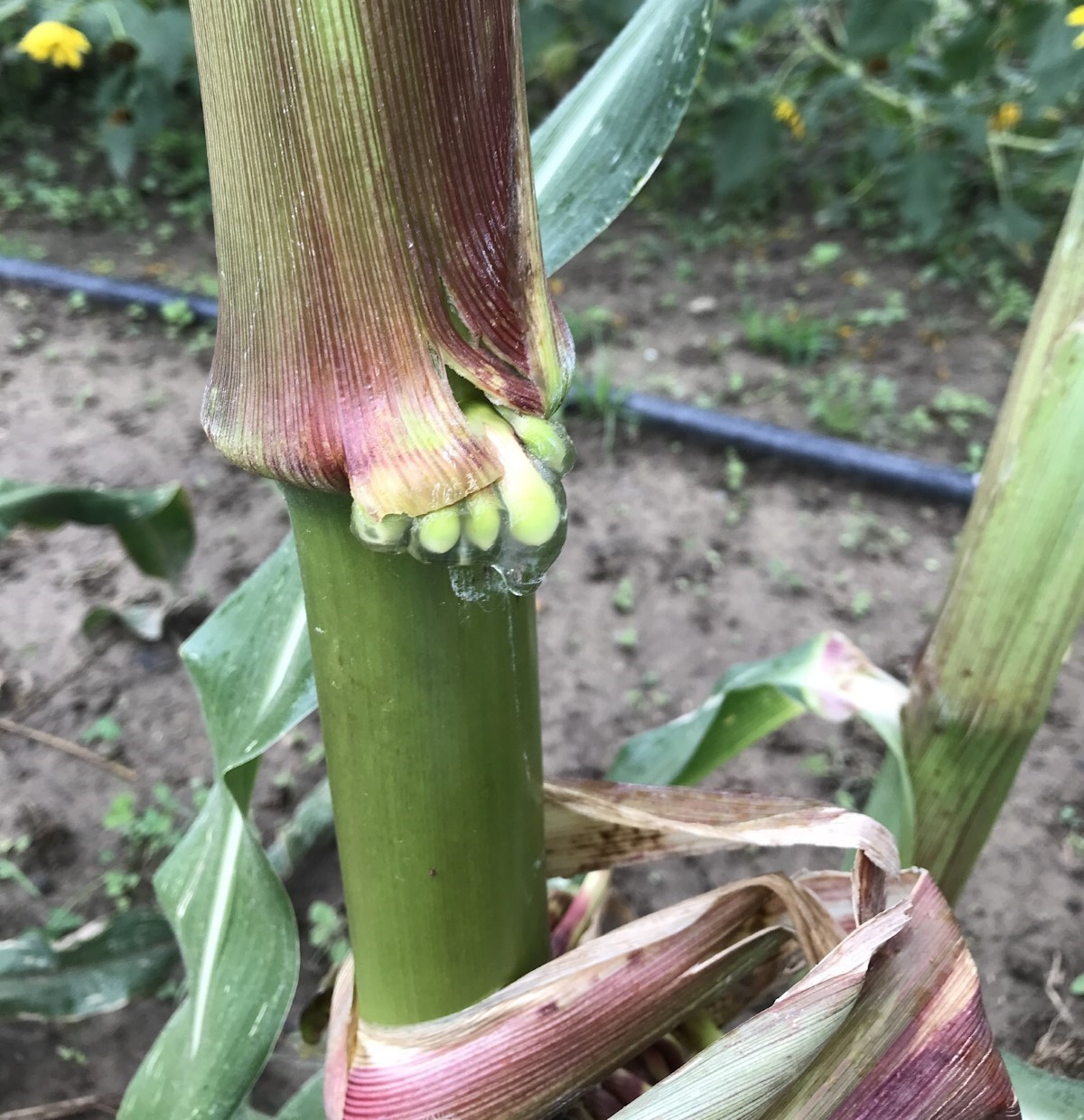


**Supplementary Figure S1**: Aerial roots broken through the outer stem layer, counted as an aerial node. Mucilage coating aerial roots observed on maize landraces grown in Columbus, Ohio, USA, at the Waterman Agricultural and Natural Resources Laboratory.


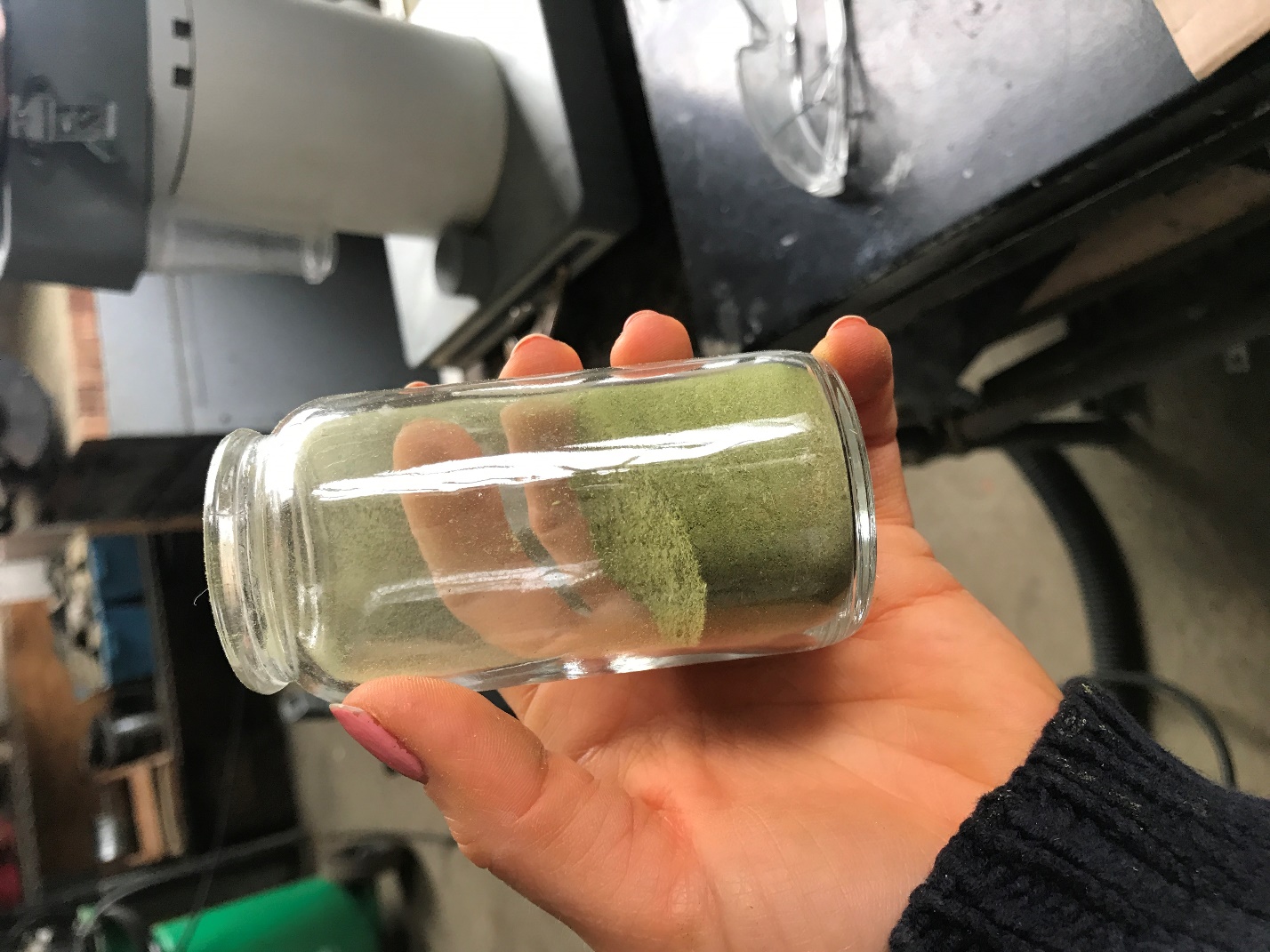


**Supplementary Figure S2:** Fine powder of ground plant biomass to be sent for ^15^N analysis after grinding sample in Udy mill.


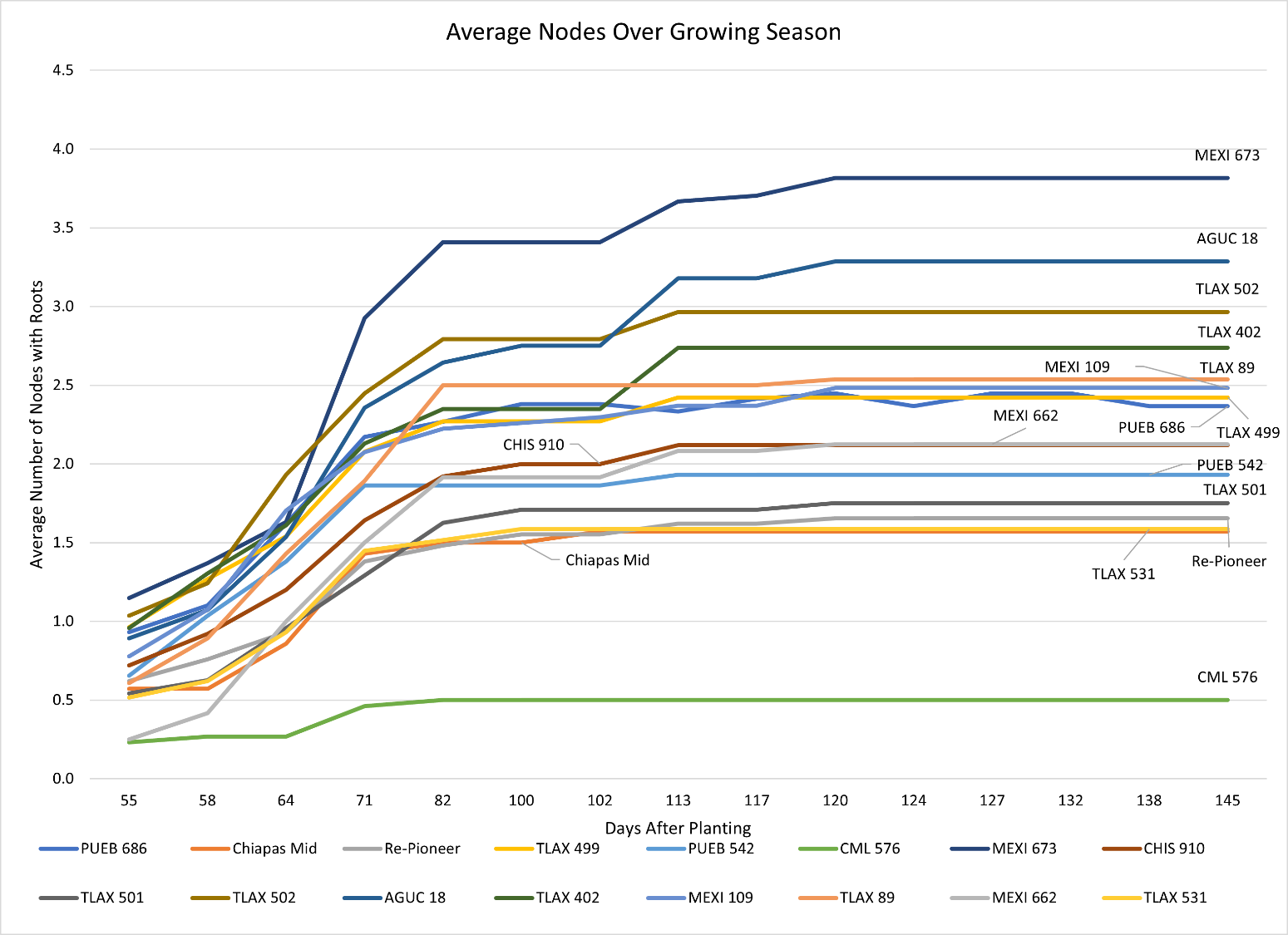


**Supplementary Figure S3:** Number of nodes with aerial roots present through the duration of the 2019 field experiment at Waterman Farm in Columbus, Ohio, USA. The nodes used in this average calculation did not include the two lowest nodes that were at or slightly above the soil surface; this resulted in lower averages per accession when compared to the 2020 experiment. The plot for each accession is an average across all plants within that accession. The “Re-Pioneer” accession was the control, and all other accession were Mexican landrace varieties (see Supplementary Table S1).


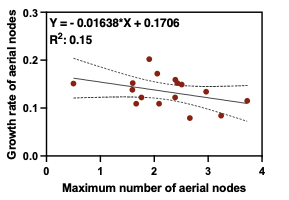


**Supplementary Figure S4:** The relationship between maximum number of aerial nodes and its growth rate. The parameters were estimated by logistic growth model. A simple linear regression model was fitted to obtain the relationship. Note that growth rate of aerial nodes refers to each node with aerial roots, not the length of each root on a node).


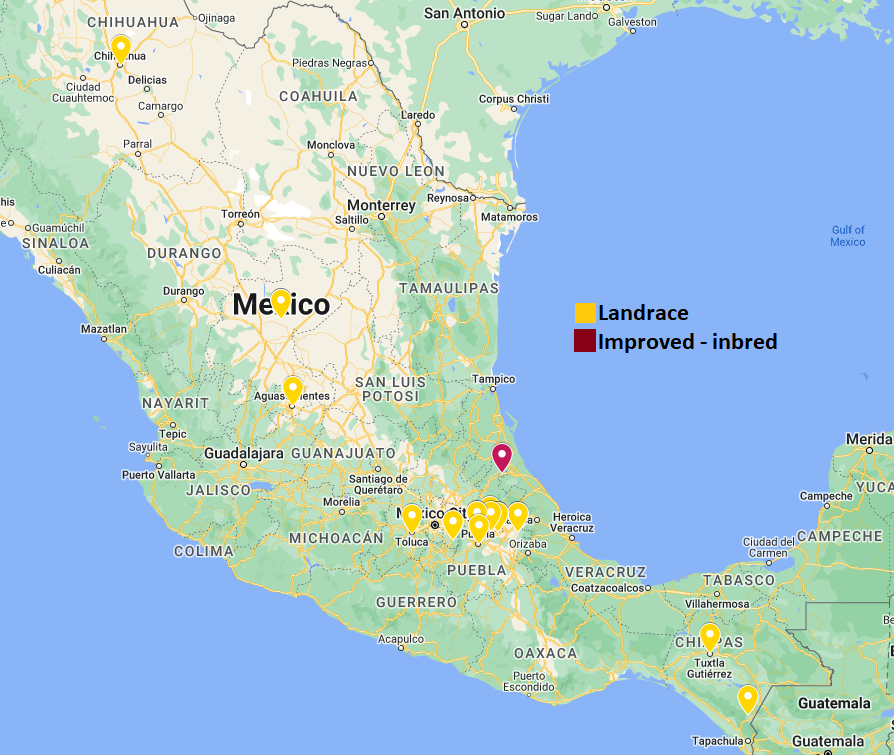


**Supplementary Figure S5:** Geographical distribution of locations of origin for *Z. mays* landraces grown in 2019 and 2020 field experiments at Waterman Farm in Columbus, Ohio, USA. One landrace sourced from Lima, Peru, not shown*.*
