# Supplementary material for: Nitrogen fixation rates and aerial root production among maize landraces
Source: Front Plant Sci. 2025 Jan 28;16:1502884. doi: 10.3389/fpls.2025.1502884 (PMC11811074; doi:10.3389/fpls.2025.1502884)
Supplement: Supplementary file 2 [file Table1.docx]

Supplementary Material: Tables

**Supplementary Table S1:** Maize accessions planted for the 2019 and 2020 field experiments in Columbus, Ohio, USA at the Waterman Agricultural and Natural Resources Laboratory.

| **Year Planted at OSU** | **Plant Name** | **Full Name / Plant ID** | **Landrace / Commercial** | **Elevation (m)** | **Origin** | **Sourced From** |
| --- | --- | --- | --- | --- | --- | --- |
| 2019 | Chiapas Mid | NA | Landrace | ~1550 | Chiapas, Mexico | H. Perales |
| 2019 | CHIS 910 | CIMMYTMA 29860 | Landrace | 2824 | El Porvenir, Chiapas, Mexico | CIMMYT |
| 2019 | MEXI 109 | CIMMYTMA 27131 | Landrace | 2717 | Toluca & Calputitlan, Mexico | CIMMYT |
| 2019 | MEXI 662 | CIMMYTMA 23384 | Landrace | 2505 | Amecameca, Mexico, Mexico | CIMMYT |
| 2019 | MEXI 673 | CIMMYTMA 23386 | Landrace | 2505 | Amecameca, Mexico, Mexico | CIMMYT |
| 2019 | PUEB 542 | CIMMYTMA 16446 | Landrace | 2514 | Guadalupe Victoria, Puebla, Mexico | CIMMYT |
| 2019 | PUEB 686 | CIMMYTMA 27241 | Landrace | 2514 | Guadalupe Victoria, Puebla, Mexico | CIMMYT |
| 2019 | TLAX 402 | CIMMYTMA 26934 | Landrace | 2465 | Cuapiaxtla, Tlaxcala, Mexico | CIMMYT |
| 2019 | TLAX 499 | CIMMYTMA 26982 | Landrace | 2746 | Huamantla, Tlaxcala, Mexico | CIMMYT |
| 2019 | TLAX 501 | CIMMYTMA 26940 | Landrace | 2514 | Huamantla, Tlaxcala & Ermenegildo Galeana, Tlaxcala Mexico | CIMMYT |
| 2019 | TLAX 502 | CIMMYTMA 26941 | Landrace | 2465 | Cuapiaxtla & Cuapiaxtla, Tlaxcala, Mexico | CIMMYT |
| 2019 | TLAX 531 | CIMMYTMA 26931 | Landrace | 2465 | Cuapiaxtla & Cuapiaxtla, Tlaxcala, Mexico | CIMMYT |
| 2019 | TLAX 89 | CIMMYTMA 19338 | Landrace | 2310 | Huamantla, Tlaxcala, Mexico | CIMMYT |
| 2019, 2020 | AGUC 18 | CIMMYTMA 23851, PI 515138 | Landrace | 2009 | Aguascalientes, Mexico | CIMMYT & USDA |
| 2020 | CHIH 148 | CIMMTYMA 6769, PI 484422 | Landrace | 1413 | Chihuahua, Mexico | CIMMYT & USDA |
| 2020 | CIM 23370 | CIMMYTMA 23370, PI 515512 | Landrace | 2465 | Tlaxcala, Mexico | USDA |
| 2020 | CIM 24046 | CIMMYTMA 24046, PI 515516 | Landrace | 2465 | Tlaxcala, Mexico | USDA |
| 2020 | CIM 8562 | CIMMYTMA 8562, PI 487802 | Landrace | 2286 | México, Mexico | USDA |
| 2020 | LIMA 13 | CIMMYTMA-009032, PI 485347 | Landrace | 223 | Lima, Peru | USDA |
| 2020 | MEXI 212 | PI 484591 | Landrace | 2500 | México, Mexico | USDA |
| 2020 | PUEB 402 | PI 485070 | Landrace | 2229 | Puebla, Mexico | USDA |
| 2019 | Re-Pioneer | NA | Improved | NA | Buffalo Seed Co. | Buffalo Seed Co. |
| 2019 | CML 576 | CIMMYTMA 31684 | Improved - inbred | 176 | Puebla & Agua Fria, Mexico | CIMMYT |
| 2020 | Conventional | 107 day non-GMO hybrid | Improved - hybrid | NA | NA | R. Minyo |
| 2020 | Sunflower | Mammoth Russian | Heirloom | NA | Ferry-Morse (brand) | Lowes, Columbus OH |

**Supplementary Table S2:** Comparison of the climate data during the growing season in locations where the maize accessions were grown. Growing season for 2019 was May through September, and for 2020 was June through September. Data obtained from weather station located at Waterman Agricultural and Natural Resources Laboratory, Columbus, Ohio, USA and the Worldclim database was used to access historical climate data for the general regions of the center of origins of the accessions grown.

| **Region (For Growing Season Only):** | **2019 Average High Temp (°C)** | **2020 Average High Temp (°C)** | **2019 Average Low Temp (°C)** | **2020 Average Low Temp (°C)** | **2019 Average Temp (°C)** | **2020 Average Temp (°C)** | **2019 Total Rainfall (cm)** | **2020 Total Rainfall (cm)** |
| --- | --- | --- | --- | --- | --- | --- | --- | --- |
| **Waterman Farm, Columbus, Ohio** | 27.9 | 28.5 | 17.0 | 17.3 | 22.3 | 22.7 | 40.0 | 29.5 |
| **Chiapas, Mexico** | 31.3 | 31.0 | 19.8 | 19.7 | 25.5 | 25.3 | 82.2 | 73.6 |
| **Mexico, Mexico** | 26.2 | 24.9 | 17.6 | 17.5 | 21.9 | 21.2 | 61.1 | 55.8 |
| **Puebla, Mexico** | 27.1 | 26.4 | 19.2 | 19.3 | 23.1 | 22.8 | 67.6 | 66.2 |
| **Tlaxcala, Mexico** | 28.8 | 28.1 | 21.1 | 21.1 | 24.9 | 24.6 | 68.6 | 68.1 |
| **Aguascalientes, Mexico** | 29.6 | 28.6 | 21.1 | 21.1 | 25.4 | 24.8 | 87.1 | 80.9 |
| **Chihuahua, Mexico** | 27.5 | 26.1 | 19.9 | 19.8 | 23.7 | 23.0 | 74.1 | 73.6 |
| **Lima, Peru** | 29.4 | 28.8 | 20.8 | 20.3 | 25.1 | 24.6 | 47.3 | 44.0 |
